# Comparison Between K_3_EDTA and Lithium Heparin as Anticoagulant to Isolate Bovine Granulocytes From Blood

**DOI:** 10.3389/fimmu.2018.01570

**Published:** 2018-07-11

**Authors:** Shima Hassan Baien, Melissa Natalie Langer, Maike Heppelmann, Maren von Köckritz-Blickwede, Nicole de Buhr

**Affiliations:** ^1^Department of Physiological Chemistry, University of Veterinary Medicine Hannover, Hannover, Germany; ^2^Research Center for Emerging Infections and Zoonoses (RIZ), University of Veterinary Medicine Hannover, Hannover, Germany; ^3^Clinic for Cattle, University of Veterinary Medicine Hannover, Hannover, Germany

**Keywords:** granulocytes, anticoagulants, blood, extracellular traps, oxidative burst

## Abstract

Granulocytes play a key role in the defense against invading pathogens. To study granulocyte functions, the isolation of a pure and active cell population from fresh blood is required. Anticoagulants and red blood cells (RBCs) lysis used in the isolation procedure may influence cell harvest, cell marker expression, and pre-activation of cells. In this study, the influence of the anticoagulants K_3_EDTA or lithium heparin and the effect of different RBCs lysis methods on bovine granulocyte population from fresh blood of healthy cows after density gradient centrifugation were investigated. Venous blood from healthy cows was collected in K_3_EDTA and lithium heparin tubes. Density gradient centrifugation to separate granulocytes from other cells was conducted using Biocoll. Then, RBCs were lysed with hypotonic water or 0.2% sodium chloride (NaCl). Immediately after isolation, harvest, viability, size, granularity, purity, and CD11b expression as a marker for granulocytes was analyzed by flow cytometry. In addition, as a marker for activation and reactivity of the granulocytes, we stimulated cells with phorbol-myristate-acetate to evaluate the release of reactive oxygen species. Furthermore, extracellular trap (ET) formation was investigated by confocal immunofluorescence microscopy in untreated control cells and cells treated with the cholesterol-depleting agent methyl-β-cyclodextrin. We did not find a significant difference in percentage of dead cells when comparing the two anticoagulants or the different RBCs lysis methods. However, the percentage of granulocytes in the harvested population was significantly less using lithium heparin blood as anticoagulant compared to K_3_EDTA. The granulocytes harvested from lithium heparin blood and water lysis exhibited higher clumping and pre-activation of unstimulated control cells as indicated by isolation of doublet cells, increased CD11b expression, and increased oxidative burst and higher amount of ET-releasing cells. Furthermore, the combination of K_3_EDTA as anticoagulant and NaCl as RBCs lysis method revealed the lowest variability and highest difference between untreated and methyl-β-cyclodextrin-treated cells when quantifying ET formation. In conclusion, density gradient centrifugation of K_3_EDTA blood resulted in higher purity of bovine granulocytes compared to lithium heparin blood. In contrast to water lysis, NaCl lysis method is recommended to avoid pre-activation of cells which may occur during hypotonic water lysis.

## Introduction

Bovine granulocytes play an important role in the first line of defense against invading pathogens (1). Granulocytes include neutrophils, eosinophils, and basophils. However, neutrophils are the most prominent circulating granulocytes varying from 86 to 91% of total granulocytes in blood (2) depending on the health status of the individual. All granulocytes are well known to counteract against infectious agents with different mechanisms namely phagocytosis, degranulation, formation of extracellular traps (ETs), and production of reactive oxygen species (ROS) (3–5). However, to study granulocyte function in more detail, the isolation of a pure and active, but not pre-activated cell population from fresh blood is required. Different protocols for bovine granulocytes isolation are described, including gradient protocols (6–8) and biomagnetic beads method based on an anti-bovine neutrophil monoclonal antibody (9). Furthermore, there are methods using FACS solution for lysis. But as this solution contains paraformaldehyde the cells cannot be used for functional assays (10). Roth and colleagues described in 1981 a gradient-based method from 200 ml blood mixed with the anticoagulant solution acid citrate dextrose. The separation of neutrophils and eosinophils was done with Ficoll–Hypaque gradient centrifugation (6). This method gave a high neutrophil purity and the cells were functionally active in different activity assays, e.g., phagocytosis and migration. However, high amount of blood needed as well as long isolation time is a disadvantage in the described procedure. Another protocol is available for the isolation from 90 ml blood mixed with disodium EDTA. After a first partial purification of a granulocyte-enriched fraction, a neutrophil and eosinophil separation by Percoll gradient resulted in approximately 97% purity of neutrophils. The functionality of isolated cells was confirmed with phagocytosis assays (7). The centrifugation time was in total 60 min including high centrifugal force steps up to 20,000 × *g*, which may lead to high pre-stimulation of cells especially artificial ET formation. Furthermore, the authors used high amount of fetal calf serum (FCS) for their assays which has shown to influence visualization of ETs (11). In 1992, a method was described using 12.5 ml blood mixed with citrate dextrose solution and then followed by a Percoll gradient to isolate neutrophils. This protocol resulted in a mean purity of 95% neutrophils with high viability despite long centrifugation steps and isolation procedure time (8).

One additional standard method to isolate granulocytes independent of the species is a Biocoll density gradient, which separates granulocytes and red blood cells (RBCs) in one pellet from other cells such as lymphocytes and monocytes. To then separate granulocytes from RBCs, different RBCs lysis solutions can be used, including isotonic ammonium chloride (NH_4_Cl) (12), hypotonic water (13), and hypotonic 0.2% sodium chloride (NaCl) (14).

The characterization of the isolated granulocyte population is possible with determination of cell surface markers. CD11b is a cell surface marker expressed by granulocytes and other leukocytes and belongs to the β2-integrin family. Therefore, CD11b antibody is often used as marker for identification of neutrophils and monocytes (15).

Importantly, the various anticoagulants, which are used for blood collection, have different mode of actions. Ethylenediaminetetraacetic acid (EDTA) and citrate chelate-free calcium ions (Ca^2+^) in plasma. Heparin prevents conversion of fibrinogen into fibrin. Therefore, an influence on the sample preparation and cell marker expression is described (16–18).

In this study, the influence of the anticoagulants K_3_EDTA and lithium heparin combined with different RBCs lysing methods on the harvest of bovine granulocyte population was investigated to establish an isolation protocol for bovine granulocytes with high purity and low pre-activation of cells. The granulocytes were isolated from fresh blood of healthy cows by density gradient centrifugation. As read-out parameters we determined cell amount, purity, viability, oxidative burst, and the release of ETs. Especially to analyze the formation of ETs, a freshly, non-activated, viable, and pure population is needed. Long incubation procedures and high centrifugal force may lead to high pre-stimulation and artificial formation of ETs. As all granulocytes use a comparable antimicrobial mechanism and are able to release ETs, the isolation protocol focused on this whole population. Some available protocols declare to isolate neutrophils, but indeed the isolated cells are granulocytes, since often neutrophils cannot be clearly separated from eosinophils and basophils.

Although initially the formation of ETs was exclusively described for neutrophils (NETs), nowadays increasing publications demonstrate that this is a general mechanism for innate immune cells including all granulocyte populations (19). Most studies on ETs have been performed on human or murine granulocytes. Also bovine neutrophils have been shown to form NETs against different pathogens like bacteria which cause mastitis in dairy cows or the parasite *Eimeria bovis* (20, 21). Interestingly, it is described that human and bovine neutrophils similarly respond to calcium ionophore and zymosan, but differently to phorbol-myristate-acetate (PMA), cytochalasin B, and concanavalin A (22). Thus, it is still unclear what kind of signaling events lead to ET formation in bovine granulocytes.

Therefore, we aimed to optimize an isolation protocol primarily for the characterization and functional assays in ET analysis and quantification for bovine granulocytes. Furthermore, it was of importance to use minimal time schedules in the protocol for efficient analysis of ET formation and other functional assays, e.g., oxidative burst at the same day.

## Materials and Methods

All listed ordering numbers (oNo.) are valid in Germany and can vary in other countries depending on the company. Procedure time is indicated in the Section “Materials and Methods” for important steps. The time description, how long a step in this protocol needs, is presented for a well-trained person. The centrifuge used in this protocol was an Eppendorf Multifuge X3R or VWR Mega star 600R.

### Blood Collection and Granulocytes Isolation

Collection of blood from healthy cows in our institute was registered at the Lower Saxonian State Office for Consumer Protection and Food Safety (Niedersächsisches Landesamt für Verbraucherschutz und Lebensmittelsicherheit, No. 12A243), and was conducted in line with the recommendations of the German Society for Laboratory Animal Science (Gesellschaft für Versuchstierkunde) and the German Veterinary Association for the Protection of Animals (Tierärztliche Vereinigung für Tierschutz e. V.) (http://www.gv-solas.de). All blood donor cows are female non-lactating and non-pregnant Holstein Friesian with a body condition score of 4–4.75. Detailed information about the cows is listed in Table S1 in Supplementary Material. The cows were all trained for blood collection and live together in one herd in a round stable. Due to frequent handling of animals, they are less stressed in contact to veterinarians and during the blood taking procedure. The blood collection needs in total a maximum time of 10 min. The time counts from going into the stable until leaving the stable. Blood was always collected in the morning around 9:00 a.m.

1. The blood donor cow was fixed in one part of the stable. The skin was disinfected with 70% ethanol. Venous blood from healthy cows was collected after puncture of the *Vena jugularis* with a cannula (STRAUSS cannula 1.80 × 43, Dispomed^®^, Gelnhausen, Germany) in 10 ml K_3_EDTA and 10 ml lithium heparin tubes (Sarstedt, oNo. 26.358 and 26.369). The blood taking process was done gently. Each tube was filled until the filling line and immediately mixed by slowly inverting the tubes.
  Pitfalls and artifacts: A fast and harsh blood taking procedure can damage cells and influence the cell number and the cell stimulation. Furthermore, a stressed animal or a too long blood taking process can result in negative effects caused by released stress hormones. Therefore, a standard blood taking process is recommended.
  Troubleshooting: Blood collection from artery should be avoided.
2. The tubes were transferred protected from light (not cooled) to the lab for the isolation. The isolation protocol started 45 min after blood collection at the latest.
3. All following pipetting steps are conducted under sterile conditions. 15 ml blood was carefully mixed with 15 ml 1× LPS-free PBS (1:2, 1× LPS-free PBS is made with 10× LPS-free PBS from Sigma Aldrich, oNo. P5493, and LPS-free water from Roth, oNo. 3255.1) in a 50 ml falcon tube (Sarstedt oNo. 62.547.254) with a sterile 10 ml plastic pipette (Sarstedt oNo. 861.254.001) avoiding air bubbles.
  Pitfalls: If LPS contamination is present in any media or buffer, as it sometimes may be found in aqua dest water reservoirs, this LPS can prestimulate the cells during isolation process.
  Artifacts: Harsh pipetting that leads to air bubbles can stress the cells and lead to pre-activation of the cells.
4. 30 ml of diluted blood was then slowly layered on the top of 15 ml Biocoll (1.077 g/ml; Merck Millipore, oNo. L6115) in a 50 ml falcon tube. This step was conducted with a 10 ml serological pipette and a pipette boy on minimum engine speed and the operating mode “release with free outlet.” The tube was positioned in a 45° angle. The diluted blood runs slowly at the wall to the top of the Biocoll layer.
  Troubleshooting: A mixing of both layers may result in less purity of the granulocyte population.
5. The gradient was centrifuged at 1,100 × *g* for 30 min at 10°C (preadjusted) in a swinging rotor without brake (deceleration = 0). Depending on the size of the rotor an extra amount of time for stopping the rotor has to be calculated. In the presented protocol the deceleration of rotor took 15 min per run.
  Pitfalls and troubleshooting: If the brake is switched on during density gradient centrifugation, the gradient may be destroyed. The usage of a fixed rotor is not possible. The gradients are immediately carefully taken out of the centrifuge and carefully transported to the working place.
6. Granulocytes and RBCs remain in the pellet. The upper layer which contains monocytes and plasma was removed with as much as possible of the Biocoll solution by a vacuum pump (Vacuubrand system with emission condenser Peltronic) with a sterile glass Pasteur pipette (Roth, oNo. 4518.1). To avoid a mixing of the monocyte layer with the granulocyte layer, first the monocyte layer is sucked off and afterward the remaining Biocoll and plasma is removed. The process is carried out with eye control.
  Pitfalls and troubleshooting: A high speed of the vacuum pump can destroy the layer or suck off cells of the granulocyte layer resulting in less cell number. Clots of cells can be sometimes seen in the Biocoll layer. These clots were removed with the vacuum pump, as they might influence the purity of granulocytes and might contain debris.
7. To harvest granulocytes, RBCs were lysed by using two different methods.
  - The first method was hypotonic water lysis by adding 20 ml sterile molecular grade LPS-free water (Roth). The tube is closed and mixed by inverting. The time of the lysis starts by adding the water into the tube and has to be stopped exactly after 20 s. Therefore, 20 ml of 2× LPS-free PBS is added with a sterile serological pipette. The lid is closed again and the sample carefully inverted for 5 s.
  - The second hypotonic lysis method was done by adding 20 ml cold 0.2% NaCl (Roth, oNo. 9265.1, solved in LPS-free water). The tube is closed and mixed by inverting. The time of the lysis starts by adding 0.2% NaCl into the tube and has to be stopped after exactly 30 s. Therefore, 20 ml cold 1.6% NaCl (solved in LPS-free water) is added. The lid is closed again and the sample carefully inverted for 5 s.
    Pitfalls and troubleshooting: Both lysis methods can influence the activation of the granulocytes and the number of living granulocytes. This is a critical point of the isolation. The strict time protocol has to be followed. A longer lysis of RBCs results in more pre-activated cells and less granulocytes.
8. The cells were centrifuged at 100 × *g* for 8 min at 4°C. Now, the brake of the centrifuge is switched on (deceleration = 9).
  Pitfalls: If the brake is switched off, the centrifuge needs longer to stop the rotor and the isolation process is extended. This can result in more pre-activated cells.
9. The supernatant was removed with a vacuum pump and a sterile glass Pasteur pipette as close as possible to the white pellet at the bottom.
  Pitfalls and troubleshooting: If less supernatant is removed, this can lead to a higher contamination with RBCs of the granulocyte fraction. But importantly, less lysis steps result in less pre-activation of cells or even cell death.
10. The lysis steps (7–9) were maximally repeated twice until the pellet became white and almost free of RBCs. Therefore, a maximum of three lysis steps was performed.
11. The pellet was resuspended in cold RPMI 1640 (Gibco, oNo. 11835063) without phenol red with a 1 ml pipette tip avoiding air bubbles and gently mixed. This step needs approximately 45 s per sample.
  Pitfalls and troubleshooting: If the pellet is not completely resuspended, the cell number cannot be precisely determined. This influences all follow-up experiments. If the cells are mixed too harsh with air bubbles, this can lead to a pre-activation of the granulocytes. A clotting of isolated cells is abnormal and should be avoided.
12. Ten µl of isolated cells in RPMI were stained with 90 µl trypan blue (Roth, oNo. CN76.1, 1:10 diluted with PBS) and counted in a Neubauer chamber. In case the cell number in the 1:10 dilution was over 100 in one big square, a 1:100 dilution (in PBS) was prepared out of the 1:10 dilution and counted. All four big squares were counted and the mean was used for the cell number calculation. This step needs approximately 5 min per sample.
  Pitfalls: Trypan blue is toxic to cells. Therefore, cells in trypan blue have to be immediately counted within 5 min. If more than 10% of cells are identified as dead, the complete experiment was stopped.
13. The cell number was adjusted to 2 × 10^6^ cells/ml and stored until usage at room temperature.

The whole isolation starting from point 3 needs around 1 h and 20 min for a trained person.

### HAEMA Fast Staining and Microscopy of Isolated Cells

1. An 8 mm glass cover slip (Thermo Scientific Ø 8 #1, oNo. CB00080RA120) was placed into a well of a 48-well plate (Greiner bio one, oNo. 677102).
2. In each well bovine granulocytes (2 × 10^5^cells/well) were seeded. Before each pipetting step the cell suspension is gently mixed with pipetting one time up and down.
  Pitfalls: Less mixing results in different amounts of cells per well, as the cells descend.
  Artifacts: The cells settle down very fast at the bottom and can start to clot. Therefore, a fast procedure and gently mixing is recommended.
3. The plate was placed into the centrifuge (VWR Mega star 600R) and centrifuged at 370 × *g* for 5 min at room temperature (20°C). Afterward plate is taken out.
4. The cover slips were carefully taken out of the well plate by using a round curved cannula and a cover slips forceps avoiding scratching of the slips (per slip 30 s are needed).
  Pitfalls: Persons should be trained to take out the cover slips, as they can easily break or flip around.
5. The slips were stained with HAEMA fast stain (DIFF Quick, Labor und Technik Eberhard Lehmann GmbH, Germany, oNo. LT005) as followed: 5 s dipping into fixation solution, 5 s dipping into staining solution I (containing Eosin), 5 s dipping into staining solution II (containing Azur), and washing in aqua dest water.
  Artifacts: A longer dipping can result in dark staining of cells with the consequence that the determination of segmented nucleus of neutrophils is difficult.
6. The sample was fixed in a wet status with the cells to the top on a glass slide and dried for 1 h at 20°C (horizontal position).
7. The samples were analyzed with a LEICA DM IL LED microscope. The slider for light rings (S80/0.30) was adjusted to 10/20. The light was set to full power and the objective HI Pm 40×/0.50 PHZ was used. The pictures were documented with a connected Leica MC120HD camera.
8. All samples were stored at 20°C.

### Granulocytes Viability Analysis

1. All samples are analyzed in duplicates. The pipetting of the assay needs 15 s per sample. The measurement process of one sample needs 1 ½ min.
2. Immediately after isolation 5 × 10^5^ cells/200 μl were stained with 5 µl propidium iodide (PI, Sigma Aldrich; oNo. P4864—10 ml, 1 mg/ml, and 1:10) in a 1.5 ml plastic tube.
3. As positive dead control cells were treated with 100 µl buffer containing 0.2% Triton X-100 (Sigma Aldrich, oNo. T8787) + 2% BSA (Sigma Aldrich, oNo. A3912) in 1× PBS. Unstained cells were used as PI negative control.
4. All samples were incubated for 10 min at room temperature in the dark.
  Pitfall and troubleshooting: Since PI is toxic for cells longer incubation times can influence the result.
5. Flow cytometry analysis was done by using Attune^®^ NxT Acoustic Focusing Flow Cytometer [Laser 488 nm (50 mW), filter BL1 = 530/30, BL2 = 574/26] with following setup:
  - Threshold was adjusted to unstained cells to remove background (i.e., noise).
  - Acquisition volume was set to 50 µl (total draw volume 100 µl), acquisition speed was set to 100 µl/min and a total of 10,000 events were recorded.
  - PI fluorescence was collected in the BL2 channel.
  - PI red fluorescence intensity was analyzed using FlowJo software version (v)10. For determination of living and dead cells, gates were set with regards to the dead control and the unstained control (live cells).
    Pitfalls: Here, all FACS analysis in one experimental set-up was analyzed with the same gate. An individual change of gates for each sample may subjectively influence the results.

### CD11b Antibody Staining

1. All samples are analyzed in duplicates.
2. 5 × 10^5^cells/100 μl were stained with 100 µl staining solution containing FITC-labeled antibody against CD11b (GeneTex, oNo. GTX43767, 0.1 mg/ml; 1:400 dilution).
3. 100 µl staining solution of FITC Mouse IgG2b (BD Biosciences, oNo. 565379, 0.5 mg/ml; 1:2,000 dilution) was used as isotype control. Unstained cells were used as negative control.
4. Cells were incubated for 30 min in the dark at room temperature.
5. Cells were washed twice with 1× PBS (step needs 2 min) and centrifuged (Heraeus Fresco 17) at 200 × *g* 4°C for 10 min.
6. Finally, the pellet was resuspended in 1 ml 1 × PBS and measured with Attune^®^ NxT Acoustic Focusing Flow Cytometer with following setup:
  - Threshold and acquisition volume same as described in case of viability assay.
  - Percentage of CD11b positive cells was analyzed using FlowJo softwarev10 by adjusting CD11b fluorescence intensity comparing to isotype control.
  - Percentages of granulocytes were analyzed based on FSC and SSC.

### ROS Production

1. All samples are analyzed in duplicates.
2. Granulocytes (5 × 10^5^ cells/250 μl) were stimulated with 0.39 µl phorbol-myristate-acetate (PMA, Sigma Aldrich, oNo. 524400-1 mg dissolved in DMSO, final concentration 25 µM) as positive control and unstimulated cells were used as negative control in 1.5 ml plastic tubes.
3. Immediately 2.5 µl of 2′,7′-dichlorofluorescin-diacetate (DCF, Sigma Aldrich, oNo. D6883, final concentration 10 µM) was added.
4. All samples were incubated at 37°C and 5% CO_2_ for 30 min.
5. Flow cytometer (Attune^®^ NxT Acoustic Focusing) analysis was done measuring mean green fluorescence intensity (X-Mean of BL-1) as relative ROS production.

### Apoptosis Assay (Annexin V/PI)

1. The assay was conducted with Annexin V Kit (Bio-Rad, oNo, ANNEX200APC).
2. All samples are analyzed in duplicates. Samples were analyzed as (a) unstained, (b) untreated, (c) positive dead control (see also granulocyte viability assay), and (d) positive apoptosis control treated with gliotoxin (Sigma Aldrich, oNo. G9893, Stock solution 1 mg/ml in DMSO, final concentration 0.1 µg/ml) and human TNFα (final concentration 10 ng/ml).
3. For each time point 5 × 10^5^ cells/200 μl were incubated in a 1.5 ml plastic tube at 37°C and 5% CO_2_ for 30 or 120 min.
4. At the end of incubation the samples are centrifuged (Heraeus Fresco 17) at 250 × *g* 20°C for 5 min and the supernatant was removed.
5. The pellet was resuspended in 100 µl binding buffer containing 5 µl Annexin V. All samples were incubated for 15 min at room temperature in the dark.
6. 500 µl binding buffer was added to each sample and samples were centrifuged (5 min, 250 h, and 20°C).
7. The supernatant was removed and 200 µl binding buffer and 5 µl PI added.
8. Flow cytometry analysis was done by using Attune^®^ NxT Acoustic Focusing Flow Cytometer as described above.
9. Threshold was adjusted to unstained cells to remove background (i.e., noise).
10. Acquisition volume was set to 50 µl (total draw volume 100 µl), acquisition speed was set to 100 µl/min, and a total of 10,000 events were recorded.
11. APC red fluorescence was collected after excitation with laser 637 (100 mW) and filter RL1 (670/14).
12. PI fluorescence was collected in the BL2 channel (see above).
13. PI red fluorescence intensity was analyzed using FlowJo software version (v)10. For determination of living and dead cells, gates were set with regards to the dead control and the unstained control (live cells).

### Induction of ETs

1. An 8 mm glass cover slip (Thermo SCIENTIFIC Ø 8 #1) was placed into a 48-well plate (Greiner bio one; 100 µl/well) and coated with Poly-l-lysine by using the manufacturer’s protocol (Sigma Aldrich, oNo. P4707-50ml) with slight modifications. Poly-l-lysine was added to the coverslips for 20 min at room temperature. Afterward the solution was aspirated and the coated cover slips were dried at room temperature overnight. The next day the cover slips were washed three times with 1 × LPS-free PBS to remove unbound Poly-l-lysine.
  Pitfalls: Without a coating the cells stick less to the cover slip. The washing of cover slips avoids pre-activation of cells by remaining Poly-l-lysine.
  Artifacts: LPS and bacterial contamination can stimulate cells to spontaneously release more ETs.
  Troubleshooting: Negative control is recommended to identify spontaneous ETs formation.
2. In each well bovine granulocytes (2 × 10^5^cells/well) were seeded. Before each well the cell suspension is gently mixed by pipetting one time up and down.
  Pitfalls: No mixing results in varying amounts of cells per well, as the cells descend. Too harsh mixing may pre-activate the cells.
3. 100 µl Methyl-β-cyclodextrin (CD, Sigma Aldrich, oNo. C4555 M = 1,331 g/mol; final concentration of 10 mM) was added as positive NET inducer, for negative control 100 µl of RPMI were added. To degrade NETs to confirm specific staining of ETs, one sample setup was incubated with 100 µl methyl-β-cyclodextrin (final concentration of 10 mM) containing 0.01 U/ml micrococcal nuclease from *Staphylococcus aureus* [MN (Stock 5,000 U/ml), N5386-50UN, Sigma Aldrich, dissolved in H_2_O].
4. The plate was centrifuged at 370 × *g* for 5 min at room temperature.
5. Cells were incubated for 120 min at 37°C 5% CO_2_. The time started by adding of stimulus to the last well.
6. Afterward fixation was done with 16% paraformaldehyde (PFA, Science services GmbH, München, Germany, oNo. E15710-S, final concentration 4%) for 15 min at room temperature.
  Pitfall: Avoid pipetting of PFA directly on the cells. Carefully pipette to the corner of well.
7. The plate was wrapped with parafilm and stored at 4°C until staining.

### Immunofluorescence Staining

Troubleshooting: Never let dry out a plate during washing steps! Shortly drying samples often show high background autofluorescence. Therefore, timing is indicated.

1. The PFA fixed cells were washed thre times with 1× PBS. A dry out of slips was prevented by fast pipetting steps in all washing steps to avoid artifacts. All staining steps were conducted inside the well plate. To wash a complete 48-well plate it takes 3 min. PBS was finally sucked off.
2. Cells were permeabilized for 5 min by adding 0.5% TritonX-100. At the end everything was sucked off with a vacuum pump (whole 48-well plate in 30 s).
3. To block unspecific binding of antibodies a blocking buffer [3% normal donkey serum (Millipore, oNo. S30), 3% cold water fish gelatin (Sigma Aldrich, oNo. G7041), 1% bovine serum albumin (BSA, Sigma Aldrich), 0.5% Tween20 (Sigma Aldrich, oNo. P1379) in 1×PBS] was added for 20 min at room temperature (whole 48-well plate in 1 min).
4. Blocking buffer was sucked off with a vacuum pump (whole 48-well plate in 30 s).
5. The first antibody was added as indicated in presence of blocking buffer for 1 h at room temperature (to pipette a whole 48-well plate takes 1 min):
  - ETs: mouse IgG2a anti DNA/histone antibody (Millipore MAB3864; 0.55 mg/ml; 1:1,000)
  - Isotype control: IgG2a from murine myeloma (Sigma Aldrich, oNo. M5534, 0.2 mg/ml; 1:100)
  - Myeloperoxidase (MPO): rabbit anti-human MPO antibody (Dako A0398; 3.2 g/l; 1:300)
  - Isotype control: Chrom pure IgG from rabbit (Jackson Immuno Research whole molecule, oNo. 011-000-003, 11.1 mg/ml; 1:1,100)
6. The cells were washed three times with 1× PBS. To wash a complete 48-well plate it takes 3 min. PBS was finally sucked off.
  Troubleshooting: Less washing results in more background.
7. The secondary antibody was added in presence of blocking buffer for 1 h at room temperature in the dark:
  Single staining ETs (histone)
  - Goat anti-mouse IgG, DyLight 488 (Thermo Scientific, oNo. 35503, 1 mg/ml, 1:500)
  Double staining MPO and NETs (histone)
  - Goat anti-mouse IgG, DyLight 633 (Thermo Scientific, oNo. 35512, 1 mg/ml; 1:500)
  - Goat anti-rabbit IgG, Alexa 488 (Thermo Scientific, oNo. A11008, 2 mg/ml; 1:500)
8. The cells were washed three times with 1× PBS. To wash a complete 48-well plate it takes 3 min. PBS was finally sucked off.
  Pitfall: Less washing results in more background.
9. The cells were washed one time with aqua dest. Aqua dest was finally sucked off (whole 48-well plate in 30 s).
10. The cells were stained with aqueous Hoechst 33342 (Sigma Aldrich, oNo. 14533, 50 mg/ml; 1:1000) in aqua dest for 10 min in the dark at room temperature (to pipette a whole 48-well plate takes 1 min).
11. The cells were washed three times with aqua dest (whole 48-well plate in 3 min).
12. The slips were taken out of the well plate carefully by using a round curved cannula and some forceps avoiding scratching of the slips (per slip 30 s is needed).
  Pitfalls: A long exposure to light results in bleaching and false negative cells. Therefore, samples should be placed as fast as possible into the dark. Persons should be trained to take out the slips, as they can easily break or flip around.
13. The sample was embedded in 3 µl ProLong Gold (Invitrogen, oNo. P36930) on top of a glass slide with cells (top down) put on glass slide and dried over night at 4°C (horizontal position).
14. The coverslips were surrounded with nail polish at the next day to avoid drying of the embedded sample.
15. All samples were stored at 4°C in dark.

### Immunofluorescence Microscopy

1. ETs were visualized using a confocal laser scanning microscope (Leica TCS SP5 AOBS) with a HCX PL APO 40× 0.75–1.25 oil immersion objective.
2. Settings were adjusted at the beginning of the session with control preparations using the isotype control and a NET positive control (CD). Saturation was checked with LEICA software.
3. On each slip three randomly selected images were taken. As all samples were conducted in duplicates, in total six images per sample were taken.
  Pitfall: As cells are often unequally distributed on the cover slip, multiple pictures should be taken.
  Artifacts: Air bubbles and inhomogeneous embedding in Prolong Gold can lead to artifacts.
4. Pictures were exported as TIFF for analysis.

### Analysis of NET Formation

Since bovine neutrophils show extreme polymorphonuclear structure, the automated analysis of NET formation as described by Brinkmann et al. was not useable for this study (16).

Analysis was done using ImageJ software (version 1.51q and k, National Institute of Health, USA) as following this protocol:

1. Fluorescence channels per image were split into green or red channel for DNA/histone complex and blue channel for nuclei.
2. After splitting, green (DyLight 488) or red (Dylight 633) fluorescence intensity per image was measured. This represented DNA/histone complexes (NET positive cells).

### Statistical Analysis

Data were analyzed by using Excel 2016 (Microsoft) and GraphPad Prism 7.04. Each experiment was performed at least three times (indicated in figure legend) with blood from different cows, and within each experiment all samples were processed in duplicates. Data are shown as mean ± SD. Differences between groups were analyzed using a one-way analysis of variance (ANOVA, family wise significance *P* < 0.05) followed by Tukey’s multiple comparison test. Differences between two groups were analyzed using one-tailed paired Student’s *t*-test. *P* values *P < 0.05 and **P < 0.01 were considered significant.

## Results

### Cell Viability Is Not Affected by the Isolation Method

**Figure 1:**
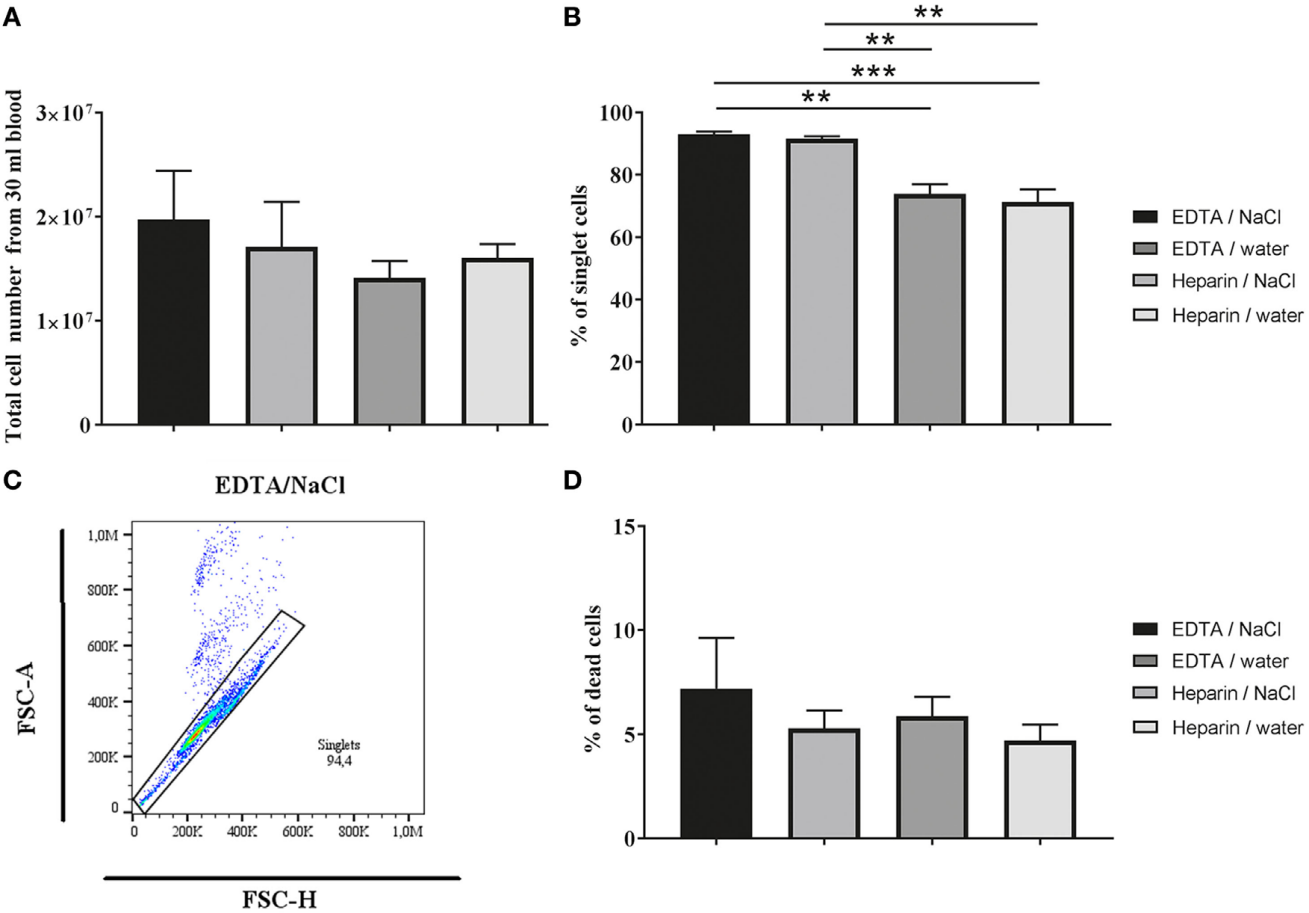
Cell harvest and dead cells after Biocoll density gradient centrifugation. **(A)** Total number of granulocytes after isolation from 30 ml blood (*n* = 5; one-way ANOVA *P* = 0. 2,606) determined by counting in a Neubauer chamber. A slight but not significant difference in the cell number was detected between the tested groups. **(B,C)** The percentage of singlet cells was analyzed by flow cytometry (*n* = 5, one-way ANOVA *P* = 0.0002). Significant difference was detected between K_3_EDTA and lithium heparin. The gating strategy is shown in **(C)**. **(D)** Percentage of dead cells from granulocytes (singlets) using propidium iodide staining analyzed by flow cytometry (*n* = 5; one-way ANOVA *P* = 0.4543). No significant difference in viability was detected. One-way ANOVA followed by Tukey’s multiple comparisons test revealed no significant difference between anticoagulants and/or erythrocyte lysis buffer in total cell number and dead cells. *P* values *P < 0.05, **P < 0.01, and ***P < 0.001 were considered significant.

As a first parameter for comparison of different granulocyte isolation methods we analyzed harvest and viability of the cells (**Figure 1**). After density gradient centrifugation and RBCs lysis, granulocytes were adjusted to the specific cell number and stained with propidium iodide to identify dead cells by FACS analysis. No significant difference was detected in total cell number (Figure 1A; one-way ANOVA *P* = 0.2606) and percentage of dead cells (Figure 1D; one-way ANOVA *P* = 0.4543) when comparing the anticoagulants K_3_EDTA and lithium heparin or the different RBCs lysis methods (Figure 1). Nevertheless, a slight tendency in cell harvest was identified: highest cell numbers were reached with K_3_EDTA/water (mean 1.71 × 10^7^ cells/ml) and K_3_EDTA/NaCl isolation method (mean 1.97 × 10^7^ cells/ml).

Importantly, when discriminating single cells (singlets) and doublets from each other based on FSC-Area (FSC-A) versus Height (FSC-H) gating (Figure 1C), a significant difference in amount of singlets was identified between the K_3_EDTA and lithium heparin group independent of the RBCs lysis method (Figure 1B; one-way ANOVA *P* = 0.0002). The data indicate that heparin group shows high amount of clotting cells leading to doublet formation. Therefore, all data in the main manuscript are presented as singlet analysis only. For comparison, all graphs including doublets and singlets are presented in supplemental material as total cell analysis (Figure S1 in Supplementary Material).

### Anticoagulants and RBCs Lysis Methods Influence Granulocyte Morphology in Mean Size and Percentage of Granulocytes

**Figure 2:**
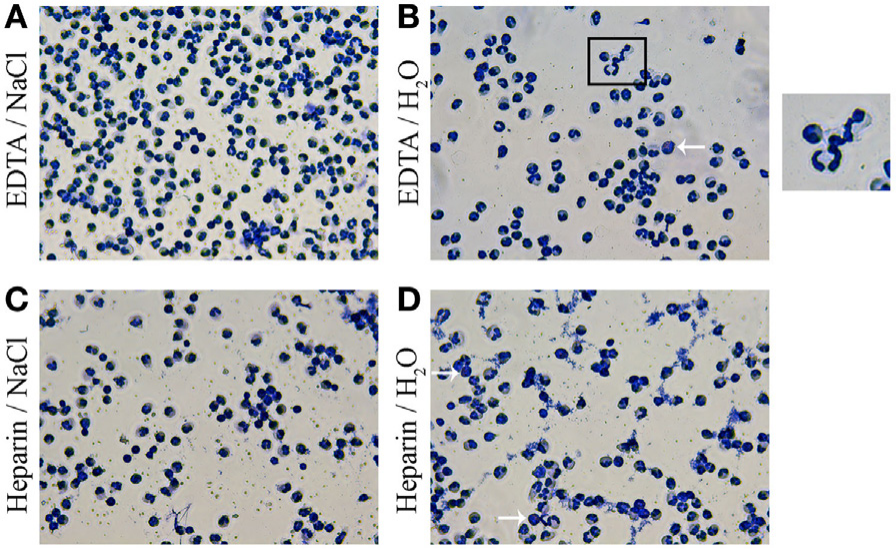
Morphology of harvested cells with HAEMA fast stain. Harvested cells were centrifuged on cover slips and stained with HAEMA fast stain (DIFF Quick). Samples were analyzed by light-microscopy. Representative pictures are shown. Independent of the anticoagulants and red blood cells (RBCs) lysis the granulocyte population after EDTA/NaCl **(A)** and lithium heparin/NaCl **(C)** isolation are presented mainly consist of polymorphnuclear granulocytes (neutrophils). Arrows indicate eosinophils with different (dark reddish) granule staining. More clotting of the granulocytes are detected in samples after lithium heparin/H_2_O isolation **(D)**. A zoom of the morphology of granulocyte segmented nucleus is shown in **(B)** and demonstrates the high variability of nucleus segmentation.

**Figure 3:**
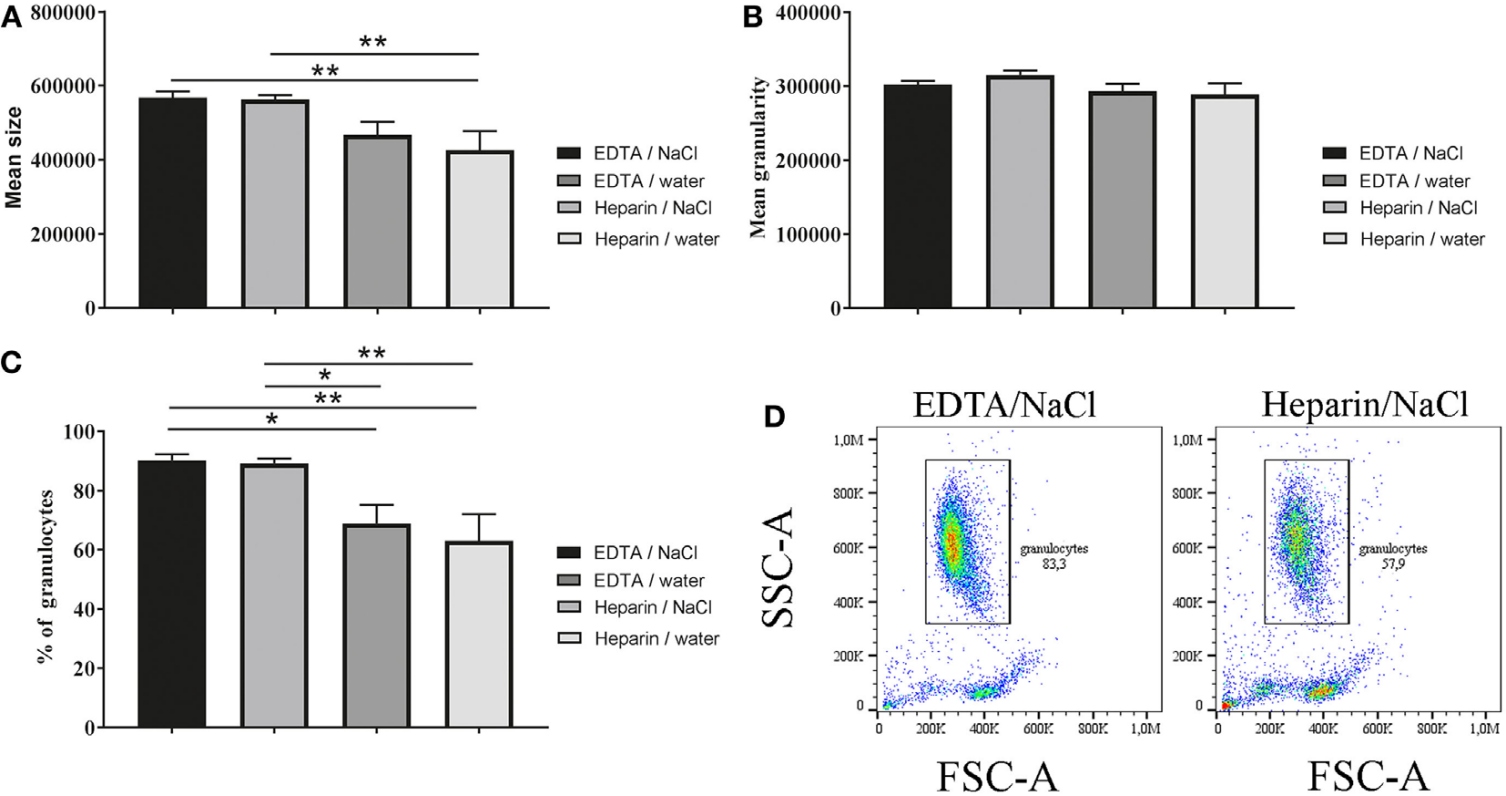
Morphology (size and granularity) of harvested cells and percentage of granulocytes. Harvested unstained cells were analyzed by flow cytometry. **(A)** Mean size of unstained cells based on forward scatter/area of intensity (FCS-A) was significant different (*n* = 5, one-way ANOVA *P* = 0.0027). **(B)** Mean granularity of unstained cells based on sideward scatter/area of intensity (SSC-A) was not significant different (*n* = 5, one-way ANOVA *P* = 0.1223). **(C)** Percentage of granulocytes was calculated based on FSC-A and SSC-A level and showed significant differences between K_3_EDTA and lithium heparin (*n* = 5, one-way ANOVA *P* = 0.0011). **(D)** Scatter blots as presented were used throughout whole experiments for subsequent data analysis. One-way ANOVA was followed by Tukey’s multiple comparisons test. *P* values *P < 0.05 and **P < 0.01 were considered significant.

As a next step, we investigated the differences of granulocyte morphology comparing the described isolation methods. A staining of isolated granulocytes with a HAEMA fast stain was used to verify and characterize the isolated population (Figure 2). Independent of the isolation a heterogeneous granulocyte population was found, with mainly neutrophils and a few eosinophils. The heteronomous segmentation of the nucleus was found in all isolated groups. Furthermore, more clotting cells were found in the heparin/water lysis isolated group (Figure 2D). By FACS analysis more detailed quantitative morphology studies were conducted: the mean size of granulocytes isolated from K_3_EDTA-treated blood was significantly higher compared to the mean size of isolated cells from lithium heparin blood (Figure 3A, one-way ANOVA *P* = 0.0027). Furthermore, the biggest mean size was measured in granulocytes after K_3_EDTA/NaCl isolation method. On the other hand, mean granularity of harvested cells was not affected by both anticoagulants and RBCs lysis method (Figure 3B, one-way ANOVA *P* = 0.1223). Based on the forward and sideward scatter (FSC-A and sideward scatter/area of intensity) analysis, the percentage of granulocytes in the harvested population was significantly less using lithium heparin blood as anticoagulants compared to K_3_EDTA blood (Figures 3C,D, one-way ANOVA *P* = 0.0011).

### Highest Percentage of CD11b Positive Cells Were Detected After K_3_EDTA/NaCl Isolation

**Figure 4:**
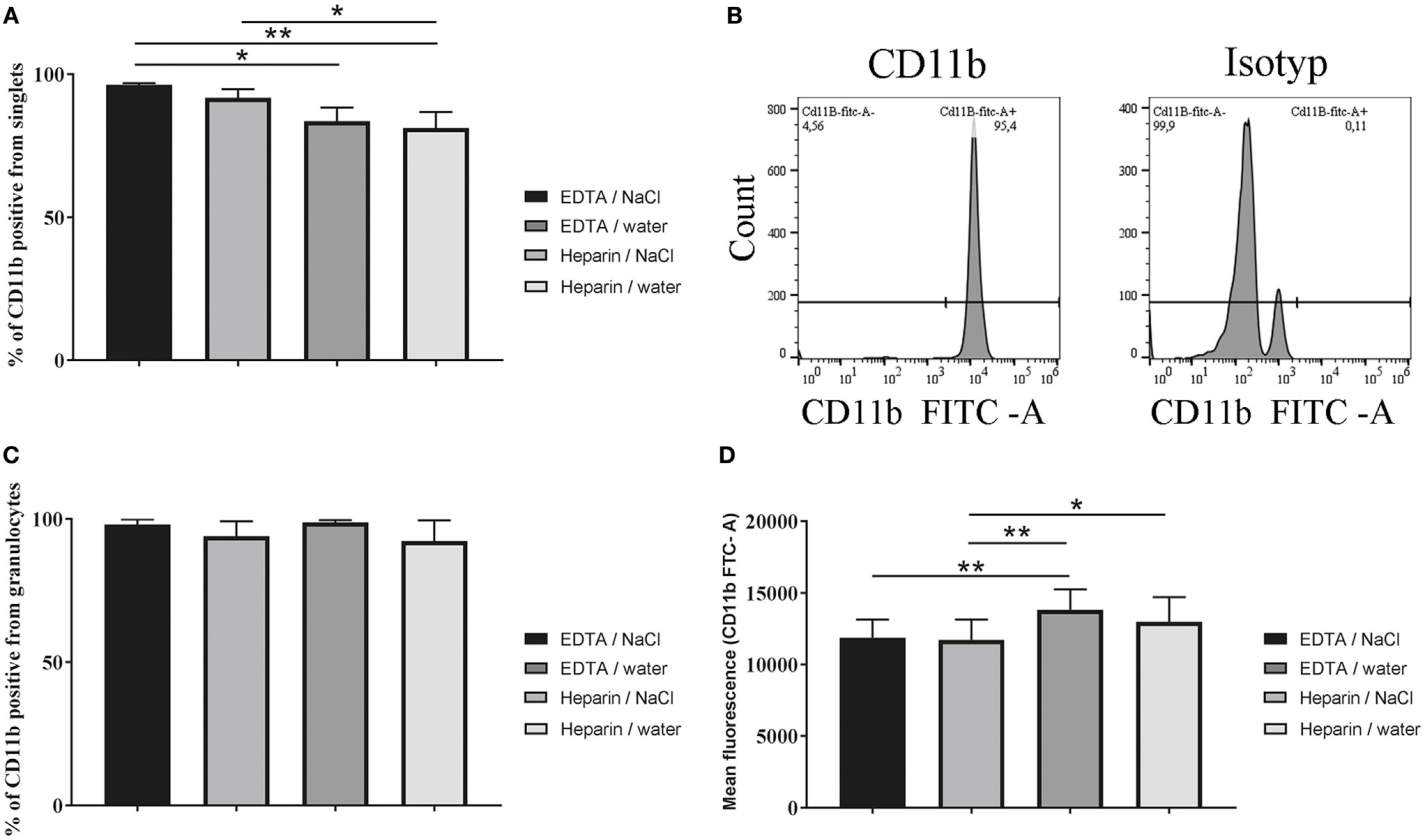
Percentage of CD11b positive cells and mean fluorescence intensity of CD11b positive granulocytes. CD11b expression of granulocytes was analyzed after isolation by flow cytometry with CD11b-specific FITC-labeled antibodies. **(A)** Percentage of CD11b positive cells from harvested cells (singlets) was significantly different (*n* = 5, one-way ANOVA *P* = 0.0033). **(B)** Scatter blots are representative for the used settings and show the count in CD11b stained samples and the isotype control after K_3_EDTA/NaCl isolation. **(C)** Percentage of CD11b positive cells from granulocytes (singlets) only after FSC/SSC gating as presented in Figure 3C was not significant different (*n* = 5, one-way ANOVA *P* = 0.3896). **(D)** By FACS the mean fluorescence intensity of CD11b expression level was analyzed on isolated granulocytes comparing the isolation methods (*n* = 5, one-way ANOVA *P* = 0.0008). One-way ANOVA was followed by Tukey’s multiple comparisons test. *P* values *P < 0.05 and **P < 0.01 were considered significant.

To analyze the isolated cells more in detail, CD11b was stained as a marker for granulocytes. A tendency for a difference in the percentage of CD11b positive cells from all harvested cells was found in cells isolated from K_3_EDTA blood compared to lithium heparin blood (Figures 4A,B, one-way ANOVA *P* = 0.0033). After K_3_EDTA/NaCl isolation the percentage of CD11b positive cells reached the highest value (96.45%) when compared to others. Since CD11b serves as marker for granulocytes these data are in good correlation with granulocyte morphology found in Figures 3C,D, confirming that the percentage of granulocytes in the harvested population was significantly less using lithium heparin blood as anticoagulants compared to K_3_EDTA blood. However, as expected almost all isolated granulocytes are positive for CD11b, independent of isolation method (Figure 4C, one-way ANOVA *P* = 0.3896). Furthermore, the mean fluorescence intensity of CD11b expression level per individual cell was tested and revealed significant lower CD11b expression in granulocytes isolated from K_3_EDTA blood compared to lithium heparin blood (Figure 4D, one-way ANOVA *P* = 0.0008). This effect was independent of the RBCs lysis method.

### Lowest Background and Highest Induction of ROS and ET Formation After K_3_EDTA/NaCl Isolation of Granulocytes

**Figure 5:**
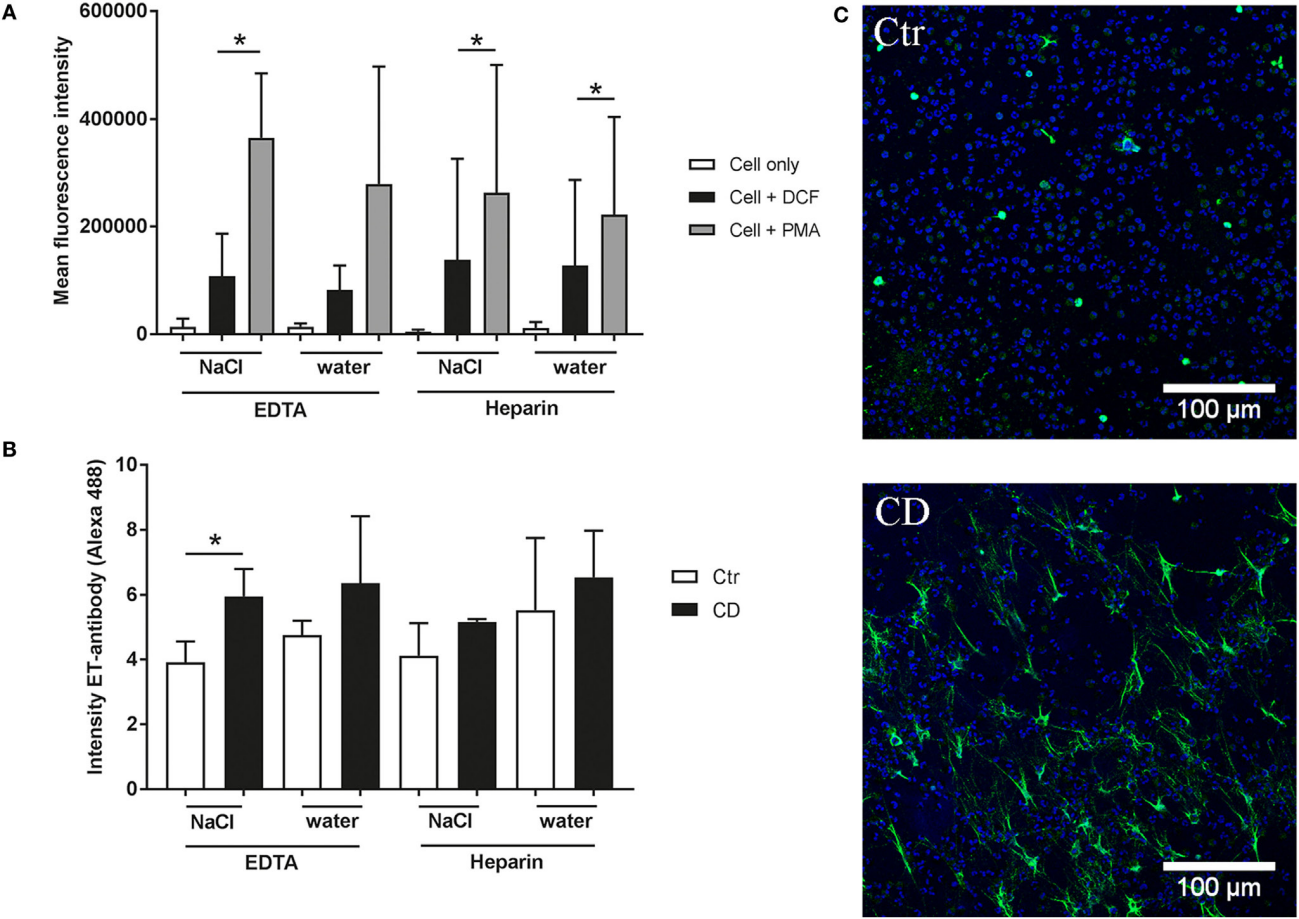
Reactive oxygen species (ROS) production and extracellular trap (ET) formation of isolated granulocytes. **(A)** Mean fluorescence intensity (X-mean) of oxidative burst (ROS production) was measured by flow cytometry (BL-1) using DCF fluorescence probe (*n* = 3). Phorbol-myristate-acetate (PMA) was used as an inducer for ROS production. A significant difference between control (cell + DCF) and PMA-treated cells was detected in 3 of 4 granulocyte isolation methods. **(B,C)** Formation of ETs was quantified based on immunofluorescence microscopy. After 2 h incubation at 37°C and 5% CO_2_ the cells were fixed. Afterward ET staining for immunofluorescence microscopy was conducted [blue = DNA (Hoechst), green = DNA/histone-1-complexes (NETs)]. Representative pictures are shown from K_3_EDTA/NaCl isolated granulocytes. The isotype control showed no ET-specific signal. Statistical analysis of ET-induction assays was conducted from three independent experiments. Negative control (Ctr) is an unstimulated control (RPMI), methyl-β-cyclodextrine (CD) was used as positive NET inducer. Per sample, 6 pictures were taken on 2 slides at predefined positions, and the fluorescence intensity of NET-positive cells was determined using ImageJ software. A significant difference between negative and positive samples was detected in K_3_EDTA/NaCl isolated granulocytes. Statistical significance was tested with one-tailed, paired Student’s *t*-test, *P* value *P < 0.05 was considered significant.

Finally, we analyzed the formation of ROS or the formation of NETs in control cells compared to a positive stimulus as a functional read-out parameter. Therefore, we stimulated the granulocytes with PMA as positive stimulus to induce the production of intracellular ROS. The results showed that PMA induces intracellular ROS production in all tested groups (Figure 5A). However, in the K_3_EDTA and water lysis group the statistical comparison between PMA treated and untreated granulocytes was not significant. The lowest background was detected in the K_3_EDTA/NaCl isolated group.

Furthermore, we investigated the ability to form ETs as response to the positive ET-inducer methyl-β-cyclodextrine (CD). The quantification is based on fluorescence intensity for ET-positive signal by immunofluorescence microscopy. We identified that CD induced ETs only in granulocytes isolated with K_3_EDTA/NaCl method with a significant difference compared to the respective negative control. This group showed the lowest variability and highest difference between unstimulated control and CD stimulated cells (Figures 5B–C; Figure S2 in Supplementary Material). Furthermore, control experiments were performed to verify formation of ETs (Figure S3 in Supplementary Material). Double staining of ETs with an antibody against myeloperoxidase as well as degradation of ET fibers with nucleases confirmed ET specificity.

## Discussion

Here, we describe a method to isolate bovine granulocytes from fresh blood for functional analysis including formation of ETs. After isolation of blood from animals, the blood was kept at room temperature, since the transport or storage on ice was described to increase cell death (23). Although already several protocols for isolation of neutrophils or granulocytes are described in literature (see Introduction), only little is known, how the isolation method of granulocytes influences the purity and at the same time the ability of the cells to release ETs. In case of human neutrophils in 1976, a standard isolation method for lymphocytes and granulocytes from blood was published (24). Interestingly, it was found that EDTA leads to a purer mononuclear cell fraction.In the present study, we compared in totally four combinations of anticoagulants and RBCs lysis buffer on the isolation of granulocytes from fresh bovine blood. As shown in Figure 1, only a slight but not significant difference in the total amount of harvested cells was detectable. The difference between K_3_EDTA/NaCl and K_3_EDTA/water is maybe due to a harsher RBCs lysis with water that could influence the granulocyte harvest in the K_3_EDTA group (Figure 1A). However, this effect is not seen in the heparin group. As it is described that heparin can lead to a clumping of cells, maybe the negative effect of water on granulocytes which is seen in the EDTA group is attenuated in heparin sampled blood, since granulocytes are partially protected against water lysis when clumping in the presence of heparin. And indeed, when terminating doublet versus singlet cell population by flow cytometry, the heparin groups showed higher amount of doublets in the harvested cell population compared to the EDTA groups (Figure 1B) confirming heparin-mediated clumping of cells. However, all tested combinations resulted in a similar number of viable cells (Figure 1D). Interestingly, for human neutrophils, an increased apoptosis rate was described after heparin incubation in a dose- and time-dependent effect (25), a finding that was not confirmed by other author (26). Effect of EDTA on apoptosis is differently described. The storage of blood mixed with EDTA resulted over time (starting already after 6 h and followed for up to 7 days) in more apoptotic cells compared to blood mixed with heparin (10). On the other hand, calcium concentrations (depletion and overload) are influencing cell death as well as the apoptosis analysis method using Annexin V (23). As an example, to study apoptosis rate of isolated granulocytes, a protocol and exemplary data are shown in our study in Material and Methods and in Figure S2 in Supplementary Material to quantify viable versus necrotic or apoptotic cells. Although apoptosis process normally requires long incubation times over more than 6 h, a slight induction of apoptosis with gliotoxin in combination with TNFα as previously by Notebaert et al. (27) can be seen already after 120 min (Figure S4 in Supplementary Material). Interestingly, analysis of heparin group including doublets showed distinct appearance of apoptotic cells, which disappeared when excluding doublet cells (Figure S4 in Supplementary Material), indicating that heparin-mediated clumping is associated with apoptosis in the heparin group confirming the results seen for human neutrophils (25).

Importantly, in our study bovine granulocytes isolated from K_3_EDTA-treated blood provided higher purity compared to lithium heparin-treated blood independently of RBCs lysis method (Figure 3). Data from a previous study also revealed that gradient separation of human neutrophils is more effective with EDTA blood compared to heparin and citrate blood (18). Interestingly, ultrasonic and microscopic examination of heparinized blood reported formation of platelet and platelet–neutrophil aggregates compared to citrated blood from same donor (28). And in another study, a gel formation of leukocytes and heparin was reported (29). All these results may explain the less effective separation using lithium heparin blood also found in our study here (Figure 3C), especially since it is well known that bovine blood contains more thrombocytes compared to human blood (30).

In a good correlation with the detected purity of granulocytes based on morphology analysis, the percentage of CD11b positive cells was significantly higher in the K_3_EDTA group compared to the lithium heparin group. When we examined percentage of CD11b positive cells from granulocyte population only, we found no difference between both tested anticoagulants. Almost all cells in the granulocyte population (more than 97.9%) were CD11b positive, (Figure 4C) confirming accordance of granulocyte identification based on granularity and CD11b expression.

When human neutrophils were isolated from blood, it was found that heparin causes marked up-regulation of CD11b and down-regulation of L-selectin on the neutrophil cell surface compared to EDTA or citrate mixture (31–33). To examine CD11b expression per individual cell in bovine blood, we tested the mean fluorescence intensity of CD11b positive granulocytes and also detected a significant higher CD11b expression in the heparin group (Figure 4D). Interestingly CD11b expression has been shown to enhance phagocytosis and oxidative burst (34).

Here, in good correlation to those data with human cells, heparin-isolated bovine control granulocytes showed a slight tendency for more ROS production, but with high variability (Figure 5A). Treatment of granulocytes with PMA significantly induced ROS production in both lithium heparin groups and the EDTA/NaCl group. This may be due to higher Ca^2+^ level in granulocytes isolated from heparinized blood which lead to increased production of ROS (35). A previous study found that induction of ROS in bovine neutrophils in response to zymosan is depending on CD11b and Ca^2+^ (4). But in case of K_3_EDTA group significant ROS production occurs only after RBCs lysis with NaCl (Figure 5A). This result is in a good correlation with data from human neutrophils were it was found that EDTA leads to a lower degree of PMA-induced respiratory burst activation compared to citrate and heparin (18). One explanation for the not significant difference in ROS production in granulocytes isolated from K_3_EDTA blood with water-based RBCs lysis may be a combination of both factors. EDTA captures calcium and, therefore, could lead to less activity. Furthermore, the water lysis method may act pre-activating to the granulocytes, so cells become exhausted (Figure 5A).

Since nothing is known about the effect of the isolation method on the formation of ETs by isolated granulocytes, we finally tested the ability to form ETs in unstimulated control cells and after incubation with the cholesterol-depleting methyl-β-cyclodextrine (CD) (36). ETs are described as an important defense mechanism against invading pathogens that is mediated by entrapment and immobilization of invading pathogens. However, when the host is not able to eliminate ETs again, an overwhelming ETs release may also contribute to detrimental effects, e.g., thrombosis or autoimmune diseases (37). The processes that mediate formation of ETs are well investigated with human neutrophils. Since bovine granulocytes have been shown to respond differently compared to human granulocytes, little is known about processes mediating ET formation in bovine cells. Therefore, improved and standardized protocols for the isolation of granulocytes are needed. In this study, we found that the isolation of granulocytes from K_3_EDTA-treated blood and RBCs lysis with NaCl led to the most effective ET-induction after CD treatment in comparison with the other isolation groups. We assume that the water treatment in both groups and/or a described formation of platelet–neutrophil aggregates in the heparin group may have led to the high or variable background ET formation in untreated cells and may have impacted the ET formation as response to a positive stimulus.

In conclusion, our results demonstrate that the purity and activity of bovine granulocytes is highly affected by the anticoagulants type and RBCs lysis buffer. This may influence outcome of results especially when higher purity of granulocytes is required. Density gradient centrifugation of K_3_EDTA blood results in higher purity of bovine granulocytes compared to heparin blood. In contrast to water lysis, NaCl lysis method is recommended to avoid pre-activation of cells (spontaneous release of ETs) which may occur during hypotonic water lysis.

## Ethics Statement

Collection of blood from healthy cows in our institute was registered at the Lower Saxonian State Office for Consumer Protection and Food Safety (Niedersächsisches Landesamt für Verbraucherschutz und Lebensmittelsicherheit, No. 12A243), and was conducted in line with the recommendations of the German Society for Laboratory Animal Science (Gesellschaft für Versuchstierkunde) and the German Veterinary Association for the Protection of Animals (Tierärztliche Vereinigung für Tierschutz e. V.) (http://www.gv-solas.de).

## Author Contributions

SB, NB, and MK-B conceived and designed the experiments. SB, ML, and MH performed the experiments. SB, ML, NB, and MK-B analyzed the data. SB, NB, and MK-B wrote the manuscript. All authors contributed to manuscript revision, read and approved the submitted version.

## Conflict of Interest Statement

The authors declare that the research was conducted in the absence of any commercial or financial relationships that could be construed as a potential conflict of interest.
