# Supplementary material for: Comparison Between K3EDTA and Lithium Heparin as Anticoagulant to Isolate Bovine Granulocytes From Blood
Source: Front Immunol. 2018 Jul 11;9:1570. doi: 10.3389/fimmu.2018.01570 (PMC6052352; doi:10.3389/fimmu.2018.01570)
Supplement: Supplementary file 1 [file data_sheet_1.PDF]

Supplementary Material

Comparison between K_3EDTA and lithium heparin as anticoagulant to isolate bovine granulocytes from blood

Shima Baien[1,2], Melissa Langer[1,2], Maike Heppelmann[3], Maren von Köckritz-Blickwede[1,2†]*, Nicole de Buhr[1,2†]*

Department of Physiological Chemistry, University of Veterinary Medicine Hannover, Hannover, Germany[1]

Research Center for Emerging Infections and Zoonoses (RIZ), University of Veterinary Medicine Hannover, Hannover, Germany[2]

Clinic for Cattle, University of Veterinary Medicine Hannover, Hannover, Germany[3]

†These senior authors contributed equally to this paper.

***Corresponding authors**

Maren von Köckritz-Blickwede

mkoeckbl@tiho-hannover.de

Nicole de Buhr

Nicole.de.buhr@tiho-hannover.de

1 Supplementary Data

none

2 Supplementary Figures and Tables

2.1 Supplementary Figures

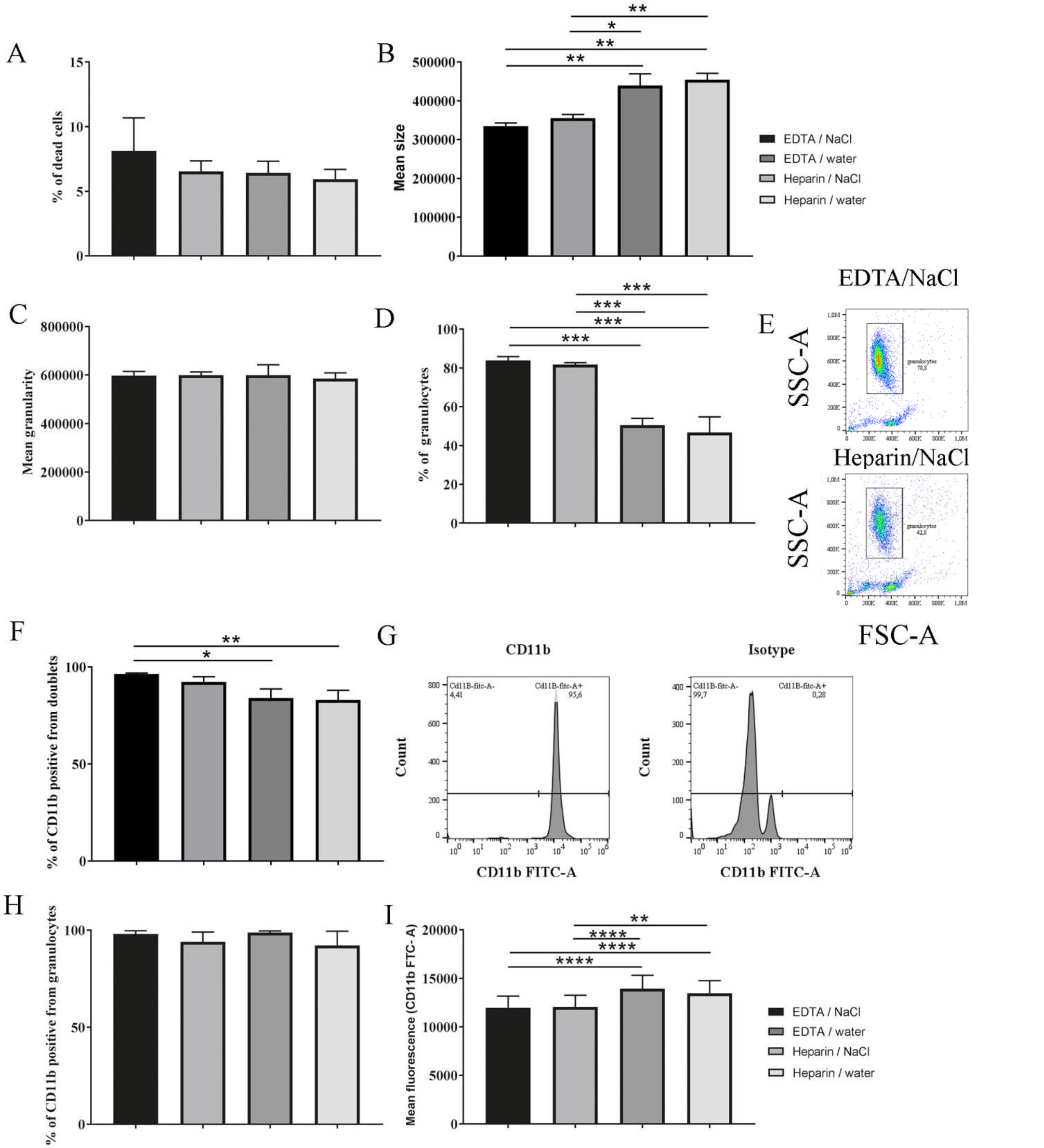

Supplementary Figure 1. All data presented in the main figures 1-3 with singlet analysis are here presented with doublet analysis. (A) Viability of isolated granulocytes (n= 5, one-way ANOVA *P*=0.5929), (B) Size of granulocytes (n= 5, one-way ANOVA *P*=0.0008), (C) Granularity of isolated granulocytes (n= 5, one-way ANOVA *P*=0.9396), (D and E) Percentage of granulocytes based on

FSC-A and SSC-A (n= 5, one-way ANOVA *P*<0.0001), (F) CD11b positive cells in all doublets (n= 5, one-way ANOVA *P*=0.0032), (G) Scatter blots are representative for the used settings and show the count in CD11b stained samples and the isotype control after K_3EDTA / NaCl isolation, (H) Percentage of CD11b positive cells from granulocytes only after FSC/SSC gating (n= 5, one-way ANOVA *P*=0.3860), (I) mean fluorescence intensity of CD11b expression level of isolated granulocytes (n= 5, one-way ANOVA *P*=0.<0.0001). *P* value **P*<0.05, ***P*<0.01, ****P*<0.001 and *****P*< 0.0001 were considered significant.

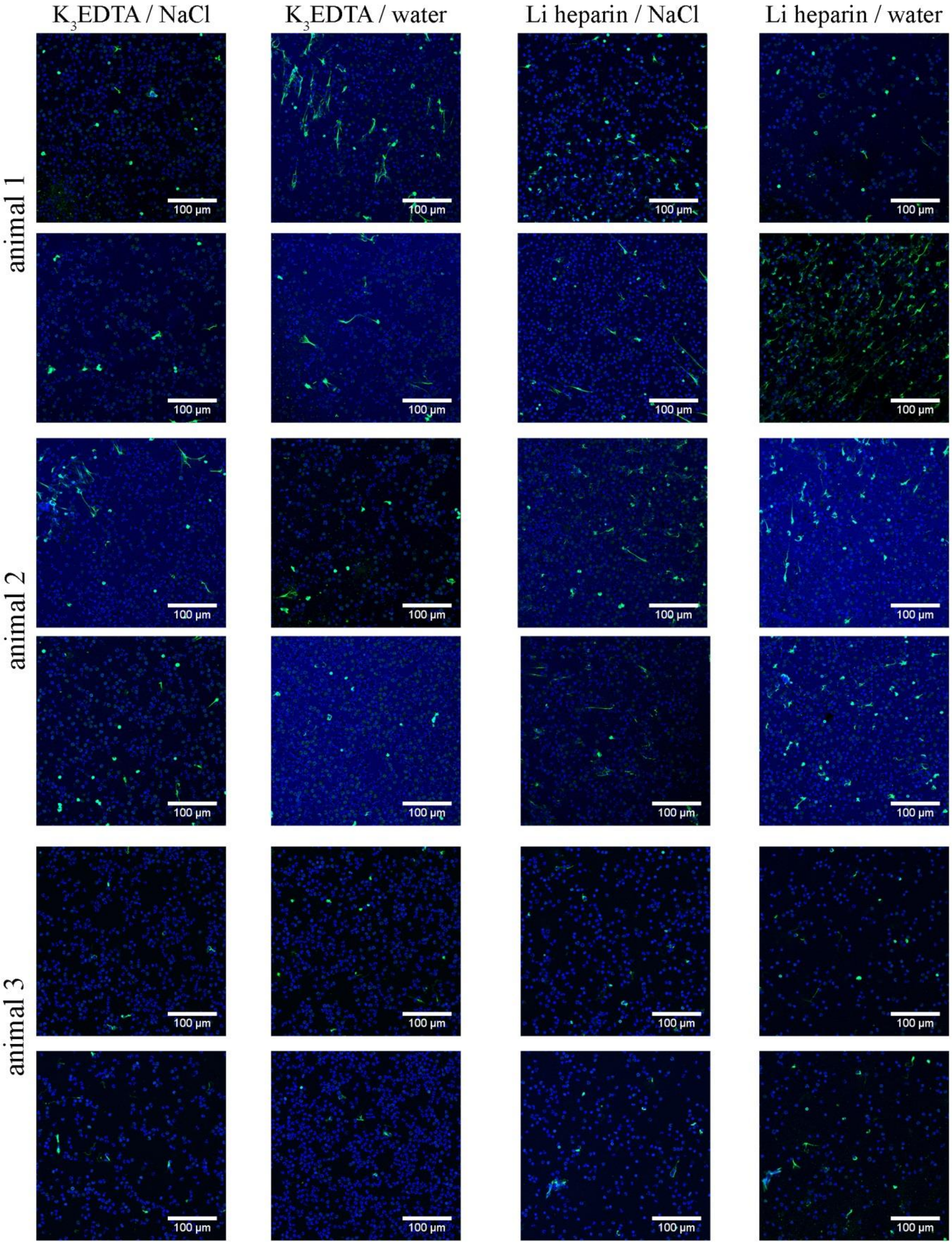

Supplementary Figure 2. Representative pictures for ET formation in unstimulated control samples. Formation of ETs was quantified based on immunofluorescence microscopy. After 2h incubation at 37°C and 5% CO_2 the cells were fixed. Afterwards ET staining for

immunofluorescence microscopy was conducted (blue = DNA [Hoechst], green = DNA/histone-1-complexes [ETs]). Two representative pictures are shown from all granulocyte isolation methods as indicated. The isotype control did not show any ET specific signal. Negative control (Ctr) is an unstimulated control (RPMI) and shows the unspecific background stimulation. Lysis methods were used as indicated at the top.

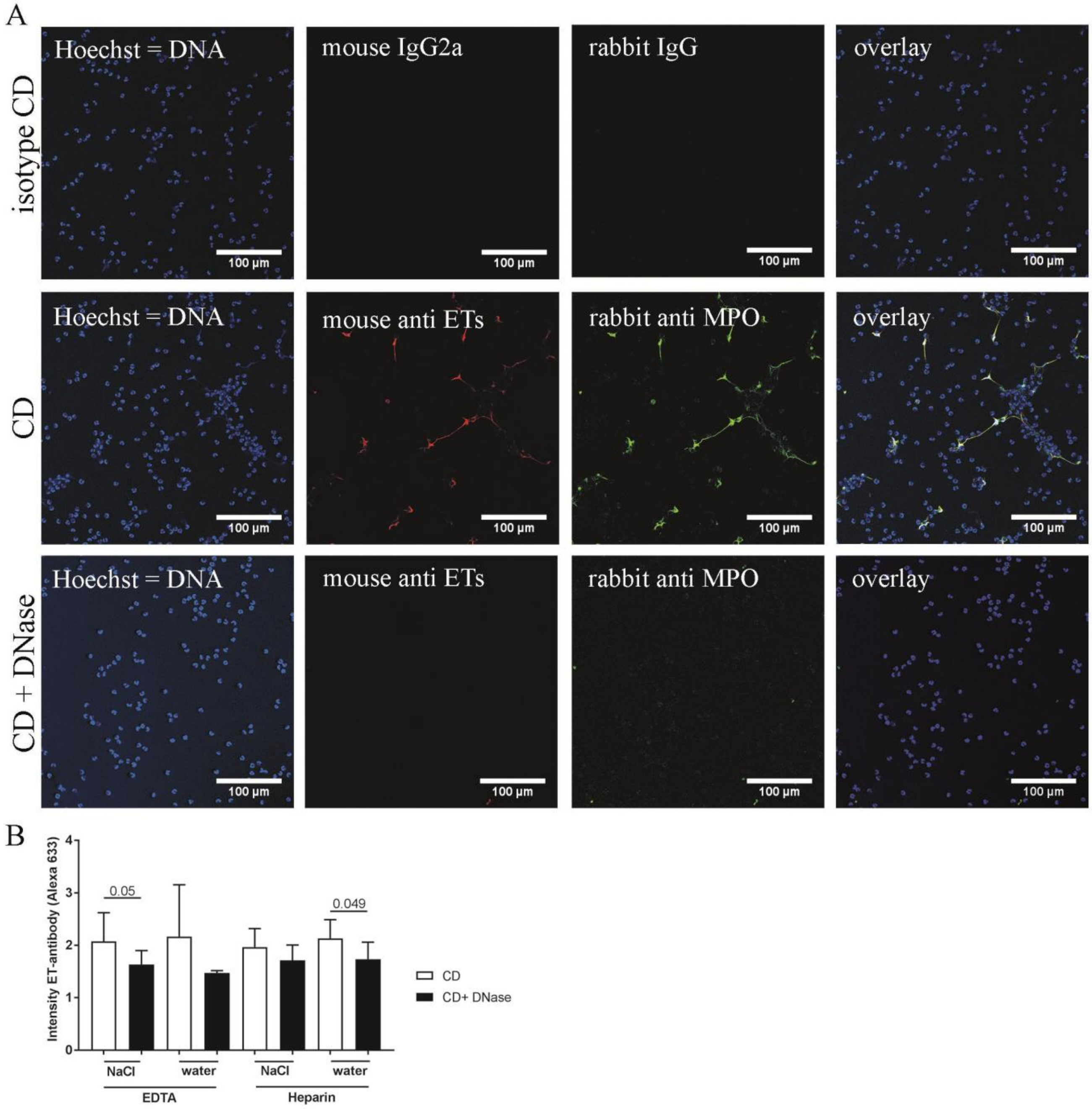

Supplementary Figure 3. Control experiments to verify formation of ETs. (1) Representative pictures for ET formation in lithium heparine / NaCl isolated granulocytes. Formation of ETs was visualized based on immunofluorescence microscopy using antibodies against myeloperoxidase in addition to ET-specific antibodies: After 2h incubation at 37°C and 5% CO_2 the cells were fixed. Afterwards ET staining for immunofluorescence microscopy was conducted (blue = DNA [Hoechst], red = DNA/histone-1-complexes [ETs], green = myeloperoxidase [MPO]). Representative pictures are shown. The isotype control did not show any ET or MPO specific signal. Cyclodextrine (CD) stimulated cells are used as positive control for ET formation. CD and DNase treated cells show a loss of ET positive signal compared to CD only, because ETs were degraded by nucleases and thus confirming ET specificity. (B) Statistical analysis of NET induction assays was conducted from 3

independent experiments. Per sample, 6 pictures were taken on 2 slides at predefined positions and the fluorescence intensity of ET-positive cells (red channel) were determined using ImageJ software. A difference between negative and positive samples was detected in K_3EDTA / NaCl and lithium heparin / water isolated granulocytes. Statistical significance was tested with one-tailed, paired Student's t-Test.

A

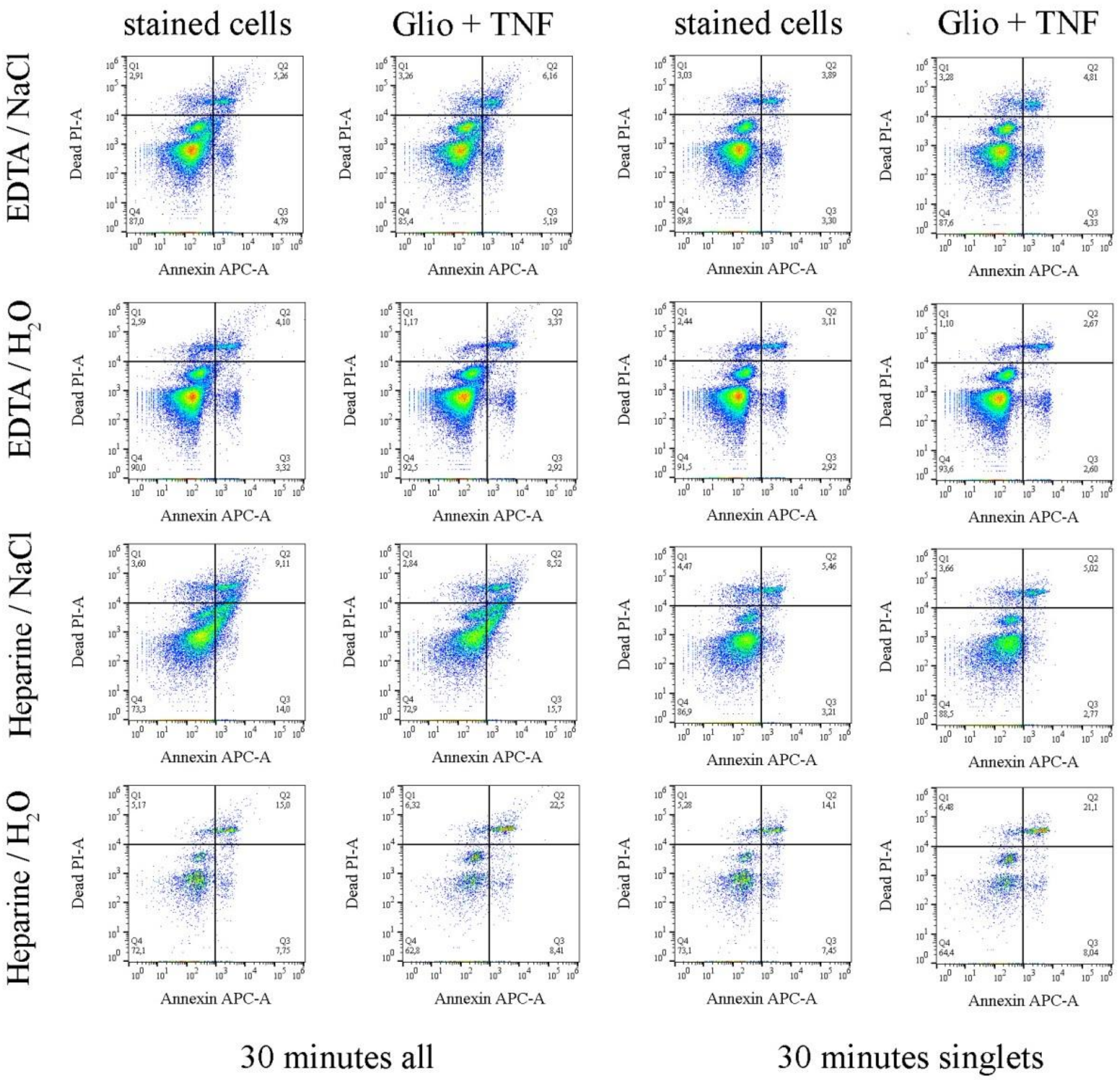

stained cells
Glio + TNF
stained cells
Glio + TNF
EDTA / NaCl
EDTA / H2O
Heparine / NaCl
Heparine / H2O
Dead PI-A
Annexin APC-A
30 minutes all
30 minutes singlets

B

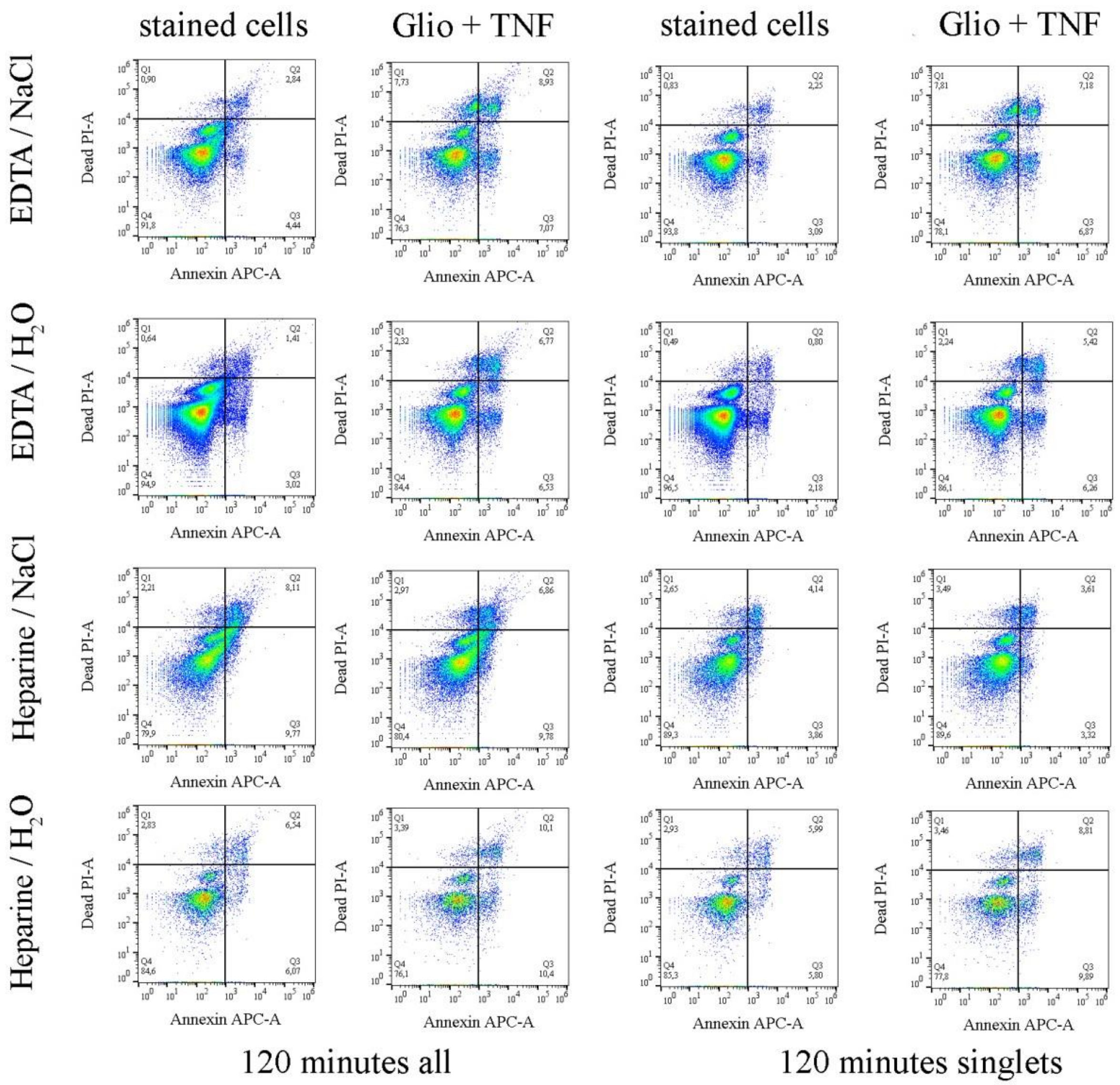
stained cells
Glio + TNF
stained cells
Glio + TNF
EDTA / NaCl
EDTA / H2O
Heparine / NaCl
Heparine / H2O
Dead PI-A
Annexin APC-A
120 minutes all
120 minutes singlets

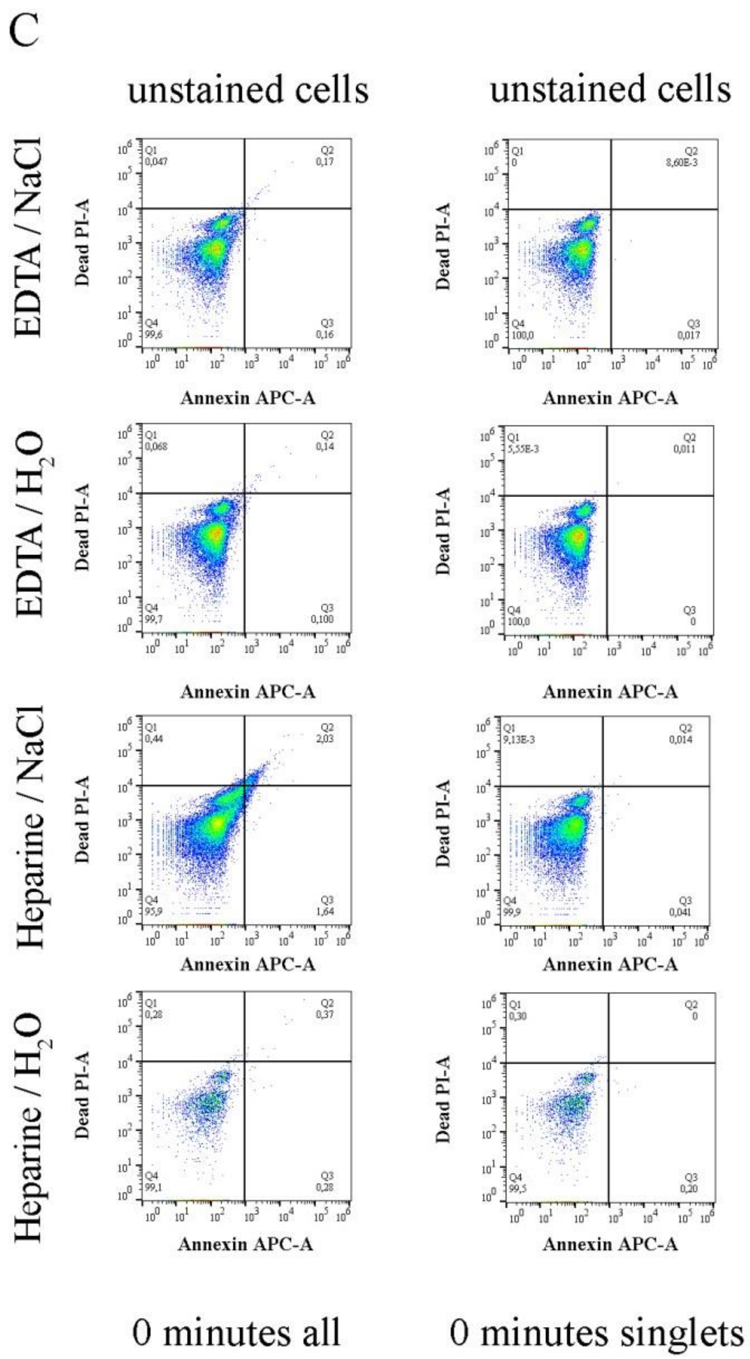

Supplementary Figure 4. Representative scatter blots for apoptosis analysis. Apoptotic cells were stained positive for with Annexin V (Annexin APC-A) and negative for propidium iodide (Dead PI-A) as shown by right lower corner (Q3) of histogram blot. Dead necrotic cells are positive for annexin V and PI (upper right corner Q2). Cells in process of undergoing necrosis at an aerly stage are positive for PI, but negative for annexin V (upper left corner Q1). Viable cells are negative for Annexin and PI (left lower corner Q4). For details see Material and Methods section in main manuscript. (A) Data shown from 30 minutes after isolation; (B) data shown from 120 minutes after

isolation; (C) data shown for unstained cells directly after isolation. Cells were incubated at 37°C and 5% CO_2, and left untreated in medium control (stained cells), or treated with 10 ng/ml TNFα (TNF) + 0.1 µg/ml gliotoxin (Glio) as apoptosis inducer. Data in the quadrants of histogram blot (Q1-4) are indicated as percentage of cells.

cow number	name	birthday	breed	lactation status	pregnant	male /female	BCS	other information
411	Kinderriegel	09.02.2011	Holstein Friesian	non lactating	no	female	4,5	Sectio 29.01.2013, 1. calf
38	Lütje	23.11.2011	Holstein Friesian	non lactating	no	female	4,25	Sectio 31.10.2013, 1. calf
202	Blacky	03.11.2011	Holstein Friesian	non lactating	no	female	4,0	Sectio 04.02.2014, 1. calf
36	Barbie	13.11.2011	Holstein Friesian	non lactating	no	female	4,25	Sectio 06.11.2013, 1. calf
271	no name	12.07.2012	Holstein Friesian	non lactating	no	female	4,25	Sectio 01.07.2014, 1. calf
38	Lütje	23.11.2011	Holstein Friesian	non lactating	no	female	4,25	Sectio 31.10.2013, 1. calf
36	Barbie	13.11.2011	Holstein Friesian	non lactating	no	female	4,25	Sectio 06.11.2013, 1. calf
372	Athene	09.01.2013	Holstein Friesian	non lactating	no	female	4,25	Sectio 26.05.15, 1. calf
411	Kinderriegel	09.02.2011	Holstein Friesian	non lactating	no	female	4,5	Sectio 29.01.2013, 1. calf
430	Rosi	04.03.2009	Holstein Friesian	non lactating	no	female	4,75	16.04.11, 1. calf
6	Flecki	30.12.2011	Holstein Friesian	non lactating	no	female	4,25	Sectio 01.06.12, 1. calf

Supplementary Table 1. Information about all blood donor cows included in this study.
